# Approach–Avoidance Conflict Paradigms in Animal and Human Studies of Anxiety—A Narrative Review

**DOI:** 10.3390/bs15111528

**Published:** 2025-11-10

**Authors:** Shijie Liu, Ziqiang Xin, Huiwen Xiao

**Affiliations:** Department of Psychology, Renmin University of China, 59 Zhongguancun Street, Haidian District, Beijing 100872, China

**Keywords:** anxiety disorders, approach–avoidance conflict, animal anxiety models

## Abstract

Anxiety disorders are among the most prevalent mental health conditions worldwide, yet their assessment and treatment have long been limited by insufficient validity. To address this challenge, researchers have increasingly sought to translate approach–avoidance conflict paradigms from animal models into human experimental tasks. This review synthesizes the translational practices of four classic paradigms, namely the conditioned conflict paradigm, the open-field test, the Morris water maze, and the elevated plus maze, and introduces a “three-level, five-dimension” evaluation framework. The framework encompasses experimental design (reproducibility and operability), construct measurement (construct validity), and applied functionality (predictive and discriminant validity). Evaluation of existing studies indicates that human translational paradigms are generally feasible, showing strengths in operability and reproducibility. These paradigms reveal behavioral patterns consistent with animal anxiety models, underscoring their translational potential. However, evidence remains largely limited to behavioral indices, with little integration of subjective, physiological, or neural measures. Predictive validity is scarcely tested, and discriminant validity is confined to broad group differences rather than clinical subtypes. Current human translational paradigms provide a useful starting point but fall short of capturing the complexity of human anxiety. Future research should strengthen ecological validity, incorporate multimodal indicators, and expand testing in clinical populations to enhance predictive and discriminant validity. Such efforts are essential for advancing these paradigms toward dynamic tracking and individualized applications in both research and clinical contexts.

## 1. Introduction

Based on the 2019 report by the World Health Organization, anxiety disorders are among the most prevalent mental health conditions worldwide, affecting an estimated 301 million individuals. These disorders substantially impair emotional regulation, cognitive functioning, and social adaptation, while also imposing significant economic and public health burdens (Wilmer et al., 2021; Konnopka & König, 2020). Despite this impact, clinical assessment still relies heavily on self-report questionnaires such as the Generalized Anxiety Disorder (GAD-7) and the Hamilton Anxiety Rating Scale (HAM-A). These instruments are prone to recall errors, social desirability bias, and cross-cultural variability, and they fail to capture the inherently dynamic nature of anxiety as an emotional state that fluctuates over time (Parkerson et al., 2015; Van de Mortel, 2008; Bettis et al., 2022). This highlights the urgent need for more objective, ecologically valid, and temporally sensitive measures of anxiety.

Historically, ethical and technical constraints have led research on anxiety disorders to rely heavily on animal models to investigate underlying neurobiological mechanisms and to screen potential anxiolytic drugs (Steimer, 2011). Animal models provide highly controlled environments in which key variables—such as neurotransmitter levels, activity in specific brain regions, or the duration of stress exposure—can be systematically manipulated (Lampis et al., 2011). However, because animals’ subjective experiences cannot be directly assessed, researchers must infer emotional states from observable behaviors. Avoidance behaviors, such as reduced exploration, restricted activity, or withdrawal from threatening contexts, are often treated as core proxies for anxiety (Ball & Gunaydin, 2022). To reliably elicit such defensive responses, experimental studies commonly employ approach–avoidance conflict paradigms (Ball & Gunaydin, 2022).

Despite the considerable advances achieved by animal anxiety models in elucidating the neural mechanisms of threat avoidance and facilitating the development of novel anxiolytic agents, several important limitations remain (Bach, 2022). On the one hand, many anxiolytic agents that demonstrate efficacy in animal models have failed in human clinical trials (Thomas, 2016), or have shown only small-to-moderate therapeutic effects in patients (Carl et al., 2020; Curtiss et al., 2017). On the other hand, human anxiety is shaped by more complex cognitive and social factors—such as catastrophizing, anticipatory processing of prolonged uncertainty, and fear of negative evaluation in social contexts—which cannot be fully captured in animal models (Grillon et al., 2019). As a result, the anxiety responses elicited by animal paradigms may differ in nature and type from those experienced by humans in real-world contexts, thereby limiting the generalizability of findings and their feasibility for clinical translation.

To overcome the limitations of animal and human discrepancies in anxiety research, recent studies have attempted to translate well-established animal paradigms into human experimental paradigms. Researchers extract behavioral tasks with clearly defined mechanisms and stable effects and adapt them into paradigms suitable for human participants, with the aim of developing novel tools for the measurement, diagnosis, and treatment of anxiety.

From an evolutionary perspective, species have developed similar threat-response strategies and neural mechanisms, which provide a foundation for translating animal anxiety paradigms based on approach–avoidance conflict models into human experimental tasks. When confronted with uncertainty or potential danger, both animals and humans balance resource pursuit against risk avoidance, often engaging in avoidance as a protective strategy (Rusconi et al., 2022; Aupperle et al., 2011). For example, a human avoiding dark alleys at night and walking along the edge of a wide road illustrates the adaptive function of the anxiety system (LeDoux et al., 2017). At heightened anxiety levels, attentional biases toward threat and avoidance responses are amplified (Grupe & Nitschke, 2013). These adaptive strategies are supported by conserved neural circuits, including the amygdala, prefrontal cortex, and hippocampus, which show striking cross-species homology (Aupperle et al., 2015; Khemka et al., 2017). This evidence further reinforces the feasibility of translating animal paradigms into human experimental tasks.

However, most human adaptations have replicated only the surface features of animal tasks, such as contexts, procedures, and indices, while lacking systematic validity assessments. Accordingly, this review pursues two primary objectives. First, it synthesizes translational research on four established paradigms, namely the conditioned conflict paradigm, the open-field test, the Morris water maze, and the elevated plus maze, with an emphasis on their theoretical foundations, design characteristics, and measurement indices. Second, it advances a “three-level, five-dimension” validity framework encompassing experimental design (reproducibility and operability), construct evaluation (construct validity), and applied functionality (predictive and discriminant validity). This framework is intended to provide a systematic basis for evaluating the strengths and limitations of existing paradigms and to guide the development of more rigorous tools for the assessment and clinical application of anxiety disorders.

## 2. Anxiety Models and Their Human Translational Paradigms

According to systematic reviews by Bourin et al. (2007) and Kumar et al. (2013), animal anxiety models based on approach–avoidance conflict fall into two broad categories: conditioned conflict paradigms and unconditioned conflict paradigms. Conditioned paradigms elicit motivational conflict by pairing reward-seeking behaviors (e.g., drinking or feeding) with aversive punishment (e.g., mild electric shock). Representative tasks include the Vogel drinking conflict test (Vogel et al., 1971) and the Geller–Seifter conflict test (Geller & Seifter, 1960), both of which emphasize precise operant control under specific reinforcement contingencies. In contrast, unconditioned paradigms do not require extensive training or external punishment; instead, they capitalize on animals’ innate aversive responses to stimuli such as heights, bright illumination, or open spaces, thereby inducing conflict between exploratory drives and threat vigilance. Classic examples include the open field test, the Morris water maze, and the elevated plus maze, which are generally regarded as more reflective of naturalistic behavior. Table 1 provides a summary of these animal paradigms and their human translational counterparts.

### 2.1. Conditioned Conflict Paradigms

The conditioned conflict paradigm elicits anxiety-related responses in animals by inducing motivational conflict between the anticipation of reward and the expectation of punishment. Typically, these paradigms begin with water or food deprivation to enhance motivational drive, followed by the introduction of aversive stimuli, such as mild electric shocks, when the animals attempt to obtain the reward. This procedure establishes a behavioral conflict between reward seeking and punishment avoidance. Representative paradigms include the Vogel conflict test and the Geller–Seifter paradigm. In the Vogel test, water-deprived rodents are allowed to drink, but drinking behavior is intermittently punished with mild electric shocks, producing an approach–avoidance conflict between water reward and shock avoidance, thereby inducing anxiety-related behavior (Vogel et al., 1971). The Geller–Seifter paradigm employs alternating schedules of positive and negative reinforcement: during unpunished phases, lever pressing yields food rewards, whereas during punished phases the same response is paired with mild electric shocks. The reduction in lever-pressing frequency during punished trials is interpreted as an index of anxiety (Geller & Seifter, 1960). Conditioned conflict paradigms provide strong experimental control and show high sensitivity to benzodiazepines, features that have led to their widespread use in studies of anxiety mechanisms and in anxiolytic drug screening (Kirlic et al., 2017).

**Figure 1:**
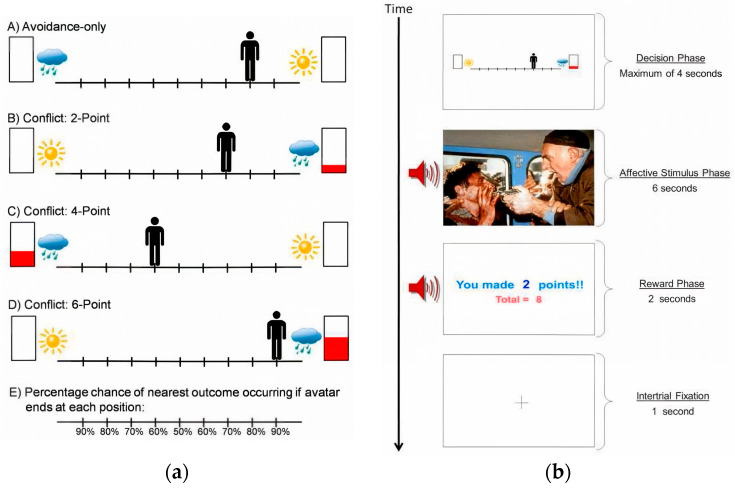
Schematic illustration of the Approach–Avoidance Conflict runway task, adopted from Aupperle et al. (2011). (**a**) Decisional conditions included within the approach–avoidance conflict task. Avoidance-only trials involve no point-reward incentives but only the possibility of viewing a positive (sun) or negative (cloud) affective stimulus. Conflict trials (2-, 4-, and 6-point levels) provide increasing point rewards for the outcome associated with the negative stimulus, whereas the competing choice yields no points but a positive stimulus. Participants move an on-screen avatar along a runway using keyboard arrow keys to indicate their relative preference between the two potential outcomes. The avatar’s final position determines the probability of each outcome (90/10% to 50/50%) across nine possible positions (−4 to +4). (**b**) Sequence of screens presented during one trial of the approach–avoidance conflict (AAC) task. Approach position = avatar’s end position on the runway relative to the negative outcome (virtual units), indexing the degree of approach versus avoidance. Initial response latency = time from cue onset to the first keypress (ms), indexing decisional hesitation. Trial structure: decision phase (≤4 s), affective-stimulus phase (6 s), reward display (2 s), and fixation (1 s).

Traditional human approach–avoidance conflict paradigms are often based on monetary rewards, with punishment typically operationalized as a reduction or loss of earnings. Such tasks generally emphasize approach behavior while neglecting avoidance, making it difficult to capture a core feature of anxiety disorders—foregoing rewards to avoid aversive emotional outcomes (Kirlic et al., 2017). To address this limitation, Aupperle et al. (2011) developed the human Approach–Avoidance Conflict (AAC) task, adapted from the animal conditioned conflict paradigm, to simulate decision-making in situations where high rewards are paired with emotional punishment (Figure 1). The task is presented in the form of a virtual runway: one side features a neutral stimulus with no reward, while the other offers varying point rewards associated with aversive stimuli, thereby inducing different levels of approach–avoidance conflict. Participants control a virtual avatar that moves laterally along the runway, with its final position determining the probability of encountering the aversive stimulus. Two core dependent variables are assessed: approach position, defined as the distance between the avatar’s final location and the aversive side, indexing avoidance tendencies; and initial response latency, defined as the delay from trial onset to the participant’s first keypress, indexing decisional hesitation.

Findings showed that approach behavior correlated positively with self-reported motivation to obtain rewards and negatively with motivation to avoid aversive stimuli, thereby supporting the task’s validity as a measure of motivational conflict. Under conflict conditions, approach behavior further increased in proportion to reward magnitude. Although participants were stratified into high- and low-anxiety groups using standardized anxiety scales, no formal between-group comparisons were conducted.

**Figure 2:**
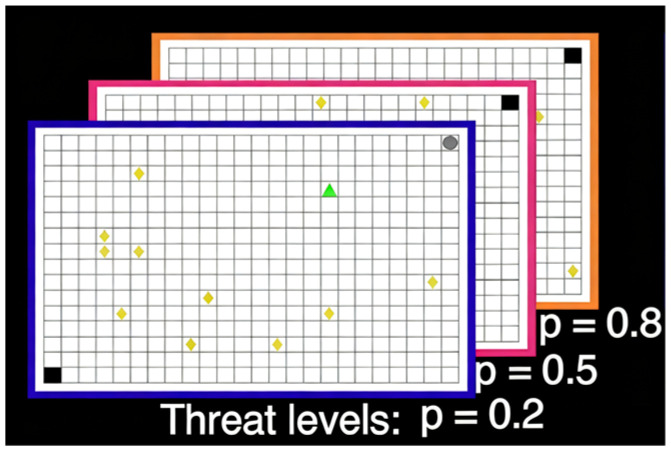
Schematic illustration of the predator Approach–Avoidance Conflict (AAC) Task, adopted from Bach et al. (2014). Participants controlled a prey avatar (green triangle) collecting reward tokens (yellow rhombi) on a 24 × 16 grid while avoiding a predator (gray circle) located in one corner. The predator remained inactive during the foraging phase but could wake up and chase the player with a threat probability of 0.2, 0.5, or 0.8. The black grid marks the designated safe zone, which the predator cannot enter. Distance from threat: mean Euclidean distance (grid units) between the prey and predator, indexing passive avoidance; Time in safe vs. threat zones: total time (s) spent in the predator’s quadrant versus the safe quadrant; Token accrual rate: number of tokens collected per second (tokens/s); Average speed: mean movement velocity of the prey avatar (grid units/s). These behavioral indices were computed over the 2 min foraging phase in each epoch and reflect canonical measures of passive avoidance and behavioral inhibition.

Building on the runway task, Bach et al. (2014) introduced a virtual grid paradigm with greater ecological flexibility to simulate contexts in which reward collection is accompanied by potential punishment (Figure 2). In this task, participants navigated a 24 × 16 grid to collect tokens while avoiding capture by randomly appearing predators; capture resulted in the loss of all accumulated tokens. The color of the grid borders represented different probabilities of predator activation, requiring participants to dynamically evaluate threat levels and decide whether to continue collecting or retreat to safety. This paradigm drew on the logic of the open-field test and the elevated plus maze, with key outcome measures including distance from the threat source, time spent in the threat quadrant, time spent in the safe quadrant, token collection rate, and movement speed outside the safe zone. Results demonstrated that as threat levels increased, participants exhibited stronger passive avoidance and behavioral inhibition. Subsequent Bayesian modeling analyses revealed that trait anxiety scores were positively associated with behavioral inhibition, with high-trait-anxious individuals exhibiting heightened sensitivity to threat (Bach, 2015).

### 2.2. Open Field Test

In 1934, Hall developed the open-field test (OFT) to evaluate rodents’ emotional responses in novel environments, which later became one of the classic animal models of anxiety. The underlying principle is that when animals are placed in a novel, open space, they simultaneously experience conflicting motivations of exploration and predator avoidance. Due to anxiety about potential threats in unfamiliar environments, animals typically avoid the central area of the arena and instead exhibit thigmotaxis—a tendency to walk along the edges (Bourin et al., 2007). This behavior serves as a core indicator of anxiety levels and is widely observed in rodents and fish (Schnörr et al., 2012; Horka et al., 2024).

**Figure 3:**
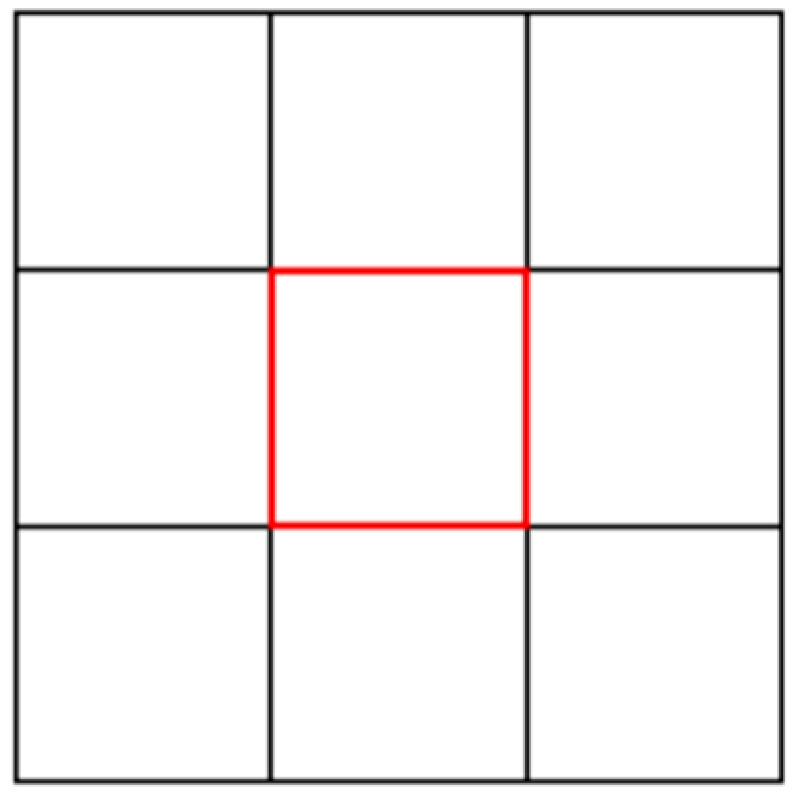
Schematic of the Animal Open-Field (OFT) Test. The red area denotes the central zone of the open field, while the surrounding area represents the peripheral zone. Time in center: total duration (s) spent in the inner zone of the arena; Center entries: number of times participants crossed from the outer region into the center; First-entry latency: time (s) from trial onset to the first entry into the center. Lower values on these measures indicate stronger avoidance of open areas (thigmotaxis). These metrics represent canonical behavioral outcomes used to quantify open-space avoidance in both animal and human versions of the OFT.

In the standard procedure, rodents are placed in a walled, unfamiliar open arena for a set period of time and allowed to explore freely, while various behaviors are recorded, including time spent in the center, number of center crossings, number of center entries, total distance traveled, defecation, and grooming (Hall, 1934) (Figure 3). Avoidance of the center is the key behavioral indicator of anxiety: animals with higher anxiety levels spend less time in the central area and display pronounced thigmotaxis along the periphery. Pharmacological studies have demonstrated that benzodiazepines and other anxiolytic drugs significantly increase time spent in the center and reduce peripheral locomotion (Prut & Belzung, 2003). Similarly, knockout of anxiety-related genes has been shown to induce comparable behavioral alterations in mice (Lotan et al., 2014).

More recently, researchers have sought to test the applicability of the OFT in humans, examining whether individuals also display thigmotaxis-like behaviors in novel environments lacking explicit threats. Walz et al. (2016) pioneered real-world human open-field experiments, instructing agoraphobic patients, healthy subjects, and high/low-anxiety-susceptibility participants to freely walk for 15 min within an open soccer field while GPS devices recorded their movement trajectories. Both route maps and heat maps revealed that all participants initially exhibited thigmotaxis. Compared with healthy controls, agoraphobic patients and high-anxiety-sensitivity individuals entered the center less frequently and showed longer latencies to their first center entry. Subsequently, Gromer et al. (2021) developed a virtual reality version of the human OFT and found that even healthy participants consistently displayed thigmotaxis. They found that healthy subjects exhibited pronounced approach–avoidance behavior, spending significantly more time in the outer regions than expected by random movement, while covering greater distances at slower speeds. The findings from both studies indicate that even in unfamiliar environments without apparent external threats, individuals spontaneously exhibit approach–avoidance behavior similar to that observed in animals.

### 2.3. Morris Water Maze

The Morris Water Maze (MWM) was originally developed to assess spatial memory and hippocampal function in rodents. Animals must locate a hidden platform within an opaque water tank using spatial cues to escape drowning. Researchers evaluate spatial memory and learning ability based on metrics such as platform search time, swimming path length, and route strategy. Results revealed that animals frequently exhibited a tendency to swim along the edges during the initial stages of the experiment, interpreted as an anxiety response triggered by environmental uncertainty (Schoenfeld et al., 2017).

Kallai et al. (2005) were the first to translate the MWM into a human version, developing the Computer-generated Arena (CGA), a desktop virtual reality platform designed to investigate human spatial navigation strategies. Participants controlled a virtual avatar from a first-person perspective using a joystick and searched for a hidden platform in a three-dimensional circular arena containing spatial landmarks. Within each trial, the platform location remained constant, while the starting position varied randomly. Performance was assessed by recording path length, latency, mean velocity, and search trajectory, allowing classification of participants’ navigational strategies. Three typical strategies were identified: thigmotaxis, visual scanning, and goal-directed navigation. Thigmotaxis represents a low-cognitive-load, emotion-driven strategy that provides boundary reference cues in the early stages of spatial learning and facilitates initial environmental orientation. However, excessive reliance on this strategy can interfere with the development of cognitive maps and hinder higher-order spatial learning.

Subsequently, Kallai et al. (2007) investigated the cognitive and emotional mechanisms of the thigmotaxis strategy in the Real Arena Maze (RAM) task. Results revealed that subjects also exhibited a similar thigmotaxis preference in the RAM task. Further analysis revealed that during the initial phase of spatial learning, thigmotaxis behavior was significantly correlated with fear levels but not with anxiety levels. Individuals scoring higher on fear scales tended to persistently explore along the edges throughout the task, overestimating the distance between the platform and the boundaries, and constructing biased cognitive maps. This finding contradicts conclusions from human open field test.

### 2.4. Elevated Plus Maze

**Figure 4:**
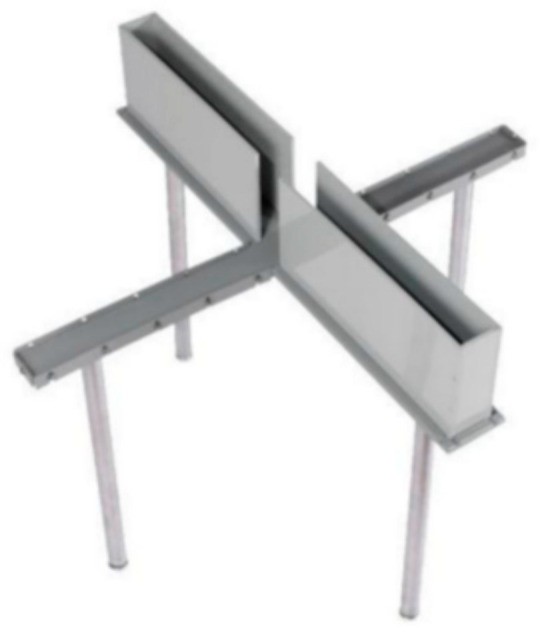
Schematic illustration of the elevated plus maze (EPM) in animals. The EPM consists of two open arms and two enclosed arms arranged in a plus shape and elevated above the ground. Participants navigate through the maze in first-person view, and behavioral indices are computed to quantify avoidance of open, potentially threatening spaces. Open-arm entries: number of times the participant enters either of the open arms; Open-arm dwell time: cumulative time (s) spent within the open arms; First-entry latency: time (s) from trial onset to the first entry into an open arm. Lower open-arm exploration and longer latencies indicate stronger avoidance behavior. These indices represent canonical outcomes of the EPM and are consistent with measures used in animal studies.

The Elevated Plus Maze (EPM) is one of the most commonly used behavioral paradigms in animal anxiety research. The apparatus features a cross-shaped configuration comprising two open arms without barriers and two enclosed arms with barriers, elevated approximately 50 cm above the ground. This design simulates the natural sense of threat associated with elevated and open spaces (as shown in Figure 4). Similar to the open-field test, the EPM elicits anxiety-related responses by inducing conflict between exploratory motivation and risk avoidance. At the beginning of the task, animals are placed on the central platform and allowed to explore freely for 5–10 min. Researchers record the number of entries into open arms, total and average dwell time per arm, whether the first entry was into an open or enclosed arm, and activity patterns in the central zone. Studies generally indicate that animals with higher anxiety levels exhibit fewer entries into open arms, shorter dwell times in open arms, and longer latency to first entry into open arms (Walf & Frye, 2007). Pharmacological studies indicate that anxiolytic drugs like diazepam significantly enhance open-arm exploration and reduce avoidance behavior (Rico et al., 2019). Mice with knockout of the hypothalamic corticotropin-releasing hormone (CRH) gene exhibit markedly reduced anxiety-like behavior (Zhang et al., 2017), demonstrating the paradigm’s sensitivity to drugs and predictive validity.

In recent years, researchers have adapted the elevated plus maze paradigm for human studies using mixed reality (MR) and virtual reality (VR) technologies (Biedermann et al., 2017; Biedermann et al., 2024). In the mixed reality version, participants physically walked on a real cross-shaped platform structure while wearing VR equipment that visually simulates being suspended over cliffs or valleys (Biedermann et al., 2017). In the virtual reality version, subjects wear VR headsets and use joysticks to navigate elevated cross mazes with varying backgrounds (Biedermann et al., 2024). In the human translation paradigm, researchers retained core behavioral metrics from animal models, focusing on key indicators such as the number of entries into open and closed arms, dwell time, and latency to first entry into open arms. Concurrently, they recorded skin conductance response, heart rate, respiratory rate, and subjective anxiety ratings. The study found that human subjects’ behavioral patterns in this scenario highly correlated with animal findings. Compared to low-anxiety subjects, high-anxiety subjects exhibited greater avoidance of the open arms in the virtual environment, manifested as fewer entries, shorter dwell times, and higher autonomic nervous system arousal.

## 3. Validity Criteria for Human Translational Paradigms

### 3.1. Existing Standards

When constructing or introducing new paradigms, their validity must first be systematically evaluated. For human anxiety experimental paradigms derived from animal models, assessing their measurement validity not only concerns the scientific rigor of the task itself but also directly impacts their applicability in both basic research and clinical settings. Validity assessment of translational paradigms should encompass two dimensions: (1) formal comparability, which examines whether the paradigm reproduces the fundamental structure of the animal task in terms of experimental context, procedures, and behavioral indices; and (2) construct-level evaluation, which examines whether the paradigm is conceptually interpretable, effectively measures the intended psychological construct, and demonstrates practical utility.

Existing evaluations have primarily focused on the first dimension—whether human anxiety paradigms formally replicate their animal counterparts. Researchers typically reconstruct the context of the animal model and adopt similar behavioral indices to build preliminary human analogues. In evaluating these paradigms, the three classic validity criteria from animal models are often directly applied, simply inverting the logic from whether animals resemble humans to whether humans resemble animals. Specifically, face validity asks whether participants display behavioral patterns resembling those of animals; predictive validity assesses whether task performance correlates with self-report measures or pharmacological interventions; and construct validity is inferred from evolutionary reasoning, i.e., whether the underlying motivational mechanisms are comparable across species.

However, these three validity criteria were originally developed to address a key limitation in animal research: animals cannot directly report subjective emotions, requiring researchers to infer emotional states indirectly through behavioral and physiological indices (Willner, 1984). In this context, the criteria are reasonable for determining whether animal models can approximate human psychopathology in terms of symptom expression, drug response, and underlying mechanisms. Yet, once anxiety paradigms are transferred from animals to humans, this framework cannot be applied uncritically. From an experimental psychology perspective, translational paradigms should be treated as independent and complete paradigms in their own right rather than as extensions of animal models. Human anxiety is far more complex than the simple threat-avoidance mechanisms simulated in animals. On one hand, humans possess the capacity for subjective reporting, self-regulation, and advanced cognitive control, while their emotional responses are further shaped by individual experiences and sociocultural influences. On the other hand, clinical diagnostic systems now differentiate subtypes of anxiety disorders, such as generalized anxiety disorder, social anxiety disorder, and panic disorder. If a translational paradigm fails to clearly establish its construct domain or to distinguish between these subtypes, both its validity and applicability will be limited.

Therefore, continuing to rely on animals as the primary reference framework risks overlooking uniquely human psychological mechanisms and fails to meet the practical research needs of clinical identification, mechanistic investigation, and intervention evaluation. Accordingly, there is a pressing need to establish a set of validity criteria specifically tailored to human translational paradigms for anxiety.

### 3.2. A Three-Level, Five-Dimension Validity Framework for Human Translational Paradigms

Validity refers to whether a test measures the psychological construct intended or the extent to which it fulfills its intended function. Therefore, evaluating the validity of an experimental paradigm requires systematic assessment based on its measurement objectives and practical functionality. Bach (2022) proposed a goal-oriented evaluation framework in their review of translational research. Building upon the three validity criteria from animal anxiety models, they further identified three clinical objectives for human translational paradigms: drug screening, clinical diagnosis, and exploration of pathological mechanisms. This framework evaluates the validity and applicability of different paradigms based on these objectives. This approach helps clarify the functional positioning of paradigms and enhances their potential for clinical application. However, this framework focuses on what the paradigm can do, without addressing the more fundamental construct measurement question: Does it scientifically measure anxiety itself? To address this gap, the present article proposes a three-level, five-dimension validity evaluation framework for human translational paradigms of anxiety. This framework seeks to restructure evaluation standards across three levels—experimental design, construct measurement, and applied functionality—thus providing both a theoretical rationale and a practical framework for future research.

**Figure 5:**
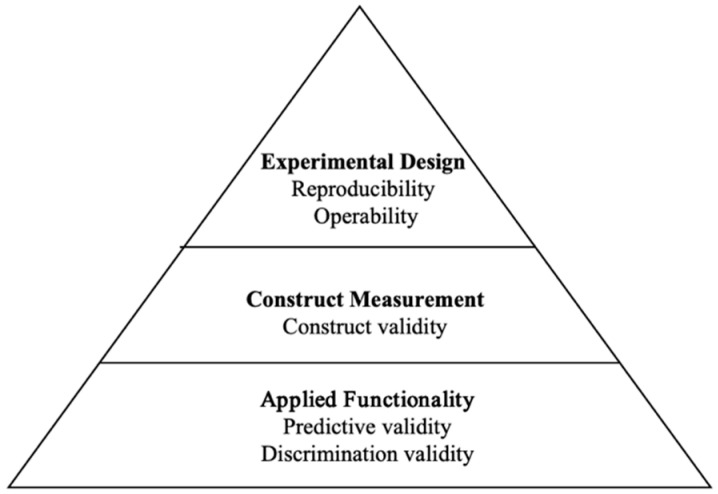
Three-Level, Five-Dimension Validity Evaluation Framework.

The framework comprises three hierarchical levels and five dimensions (Figure 5). The first level, experimental design, includes reproducibility and operability. Reproducibility refers to whether the paradigm yields consistent results across different participant samples, time points, and contexts under standardized conditions. Following established methodological conventions, an ICC ≥ 0.70 or comparable quantitative evidence from other data sources is often regarded as reflecting adequate reliability for behavioral paradigms (Hripcsak & Heitjan, 2002; Cicchetti & Sparrow, 1981). Operability emphasizes standardization of procedures and clarity in variable control. It was considered high when participant completion exceeded 90% and data exclusion remained below 10%, ensuring feasibility and standardization (Hedge et al., 2018).

The second level is the construct measurement level, which focuses on whether the paradigm genuinely measures the psychological construct of anxiety. This level includes the construct validity dimension, indicating the extent to which the paradigm’s behavioral performance, subjective experience, physiological response, and neurological indicators reflect the anxiety construct. Construct validity was considered high when converging evidence across these domains consistently reflected the anxiety construct, in line with classical definitions of convergent validity (Campbell & Fiske, 1959; Cronbach & Meehl, 1955).

The third level is the applied functionality level, which measures the paradigm’s practical value in research and clinical settings, encompassing the dimensions of predictive validity and discriminative validity. Predictive validity indicates the paradigm’s sensitivity to drug intervention effects. It was rated as high when such manipulations produced medium-to-large effects (Cohen’s d ≥ 0.50) and were replicated in at least two independent studies (Cohen, 1988).

Discrimination validity emphasizes the paradigm’s ability to distinguish between individuals, specifically whether it can differentiate between high and low anxiety levels, anxiety disorder patients and healthy individuals, and different anxiety disorder subtypes. It was judged as high when between-group differences reached at least Cohen’s d ≥ 0.50 or AUC ≥ 0.70 in two or more independent studies, representing acceptable to strong discrimination (Cohen, 1988; Hanley & McNeil, 1982). This represents the paradigm’s potential diagnostic value and feasibility for personalized interventions.

This framework represents a conceptual shift from asking whether models resemble animals to asking whether paradigms themselves demonstrate explanatory power and clinical applicability. It can be applied to systematically evaluate existing human adaptations of animal paradigms and provides an actionable theoretical and methodological foundation for the future design and optimization of anxiety paradigms.

## 4. Evaluation of Human Translational Anxiety Paradigms Based on the Three-Level, Five-Dimension Framework

### 4.1. Validity of the Approach–Avoidance Conflict Paradigm

At the experimental design level, current human AAC paradigms are predominantly implemented on computer platforms. These tasks feature clear structures and straightforward operations, requiring subjects only to control virtual characters to complete tasks. Their behavioral parameters can be used to calculate risk preferences or avoidance tendencies. Tasks can also incorporate multi-level conflict scenarios, offering strong situational controllability and parameter flexibility. Regarding replicability, both the original runway task and subsequent predator threat versions demonstrate high cross-experimental consistency. Tasks designed by Bach et al. (2014) and Bach (2015) consistently elicited behavioral inhibition responses across multiple studies, supporting this paradigm’s response stability and technical reliability under varying experimental conditions. However, no reliability indices have yet been reported in the literature, preventing a quantitative evaluation of reproducibility.

At the construct measurement level, the AAC paradigm has provided moderate construct validity supported by converging behavioral and neural evidence. Behavioral indices such as approach position and initial response latency reflect individual avoidance tendencies. In terms of neural indices, the AAC is currently the only human translational paradigm that has been used to investigate underlying neural mechanisms. Studies have shown that the runway task activates regions including the caudate, anterior insula, and dorsolateral prefrontal cortex (Aupperle et al., 2015). In the predator task, fMRI data revealed that left hippocampal BOLD activity increased linearly with threat level, and individuals with hippocampal sclerosis exhibited reduced passive avoidance and diminished inhibitory control (Bach et al., 2014). These regions are central to threat monitoring and conflict processing, reflecting cross-species consistency in underlying neural mechanisms. Notably, however, several studies have reported weak or nonsignificant correlations between AAC behavioral indices and self-reported anxiety. However, correlations between AAC behavior and self-reported anxiety are weak, and the lack of physiological evidence limits cross-modal validation, supporting only a moderate level of construct validity.

At the applied functionality level, human AAC paradigms have not yet been examined in pharmacological intervention studies, leaving their predictive validity untested. Regarding discriminant validity, some evidence indicates gender-related differences: approach behavior in men correlates positively with reward-seeking motivation, whereas women display stronger avoidance tendencies (Aupperle et al., 2011). These differences may reflect distinct mechanisms underlying reward motivation and punishment sensitivity, with male behavior more strongly influenced by reward pursuit and female behavior more readily driven by avoidance motivation. However, systematic evaluations in clinical samples are lacking, and it remains unclear whether the AAC can effectively distinguish individuals with anxiety disorders or between disorder subtypes, limiting its translational and clinical utility.

In summary, the AAC paradigm demonstrates strong performance in experimental design and provides moderate construct validity evidence from behavioral and neural measures, though evidence from subjective reports and physiological responses remains sparse. Its predictive validity has yet to be evaluated, and its discriminant validity has not been established in clinical populations. Future research should focus on validating its pharmacological sensitivity and diagnostic utility in clinical samples, clarifying the specific facets of anxiety captured by the task, and defining its scope of applicability.

### 4.2. Validity of the Open Field Test

At the experimental design level, the human open field test demonstrates strong operability and cross-population applicability due to its simple setup and intuitive task structure. Whether implemented in real-world contexts or virtual reality environments, the paradigm consistently elicits thigmotaxis (Walz et al., 2016; Gromer et al., 2021), indicating good procedural feasibility and cross-context consistency. Although convergent behavioral patterns across studies suggest acceptable reproducibility, no reliability indices have yet been reported in the literature. Moreover, current studies have not examined how contextual novelty or exposure duration may modulate thigmotaxis, which limits the paradigm’s potential application for longitudinal tracking and intervention evaluation.

At the construct measurement level, although the OFT is one of the classic animal paradigms for anxiety, there is still insufficient evidence that its human adaptation reliably elicits and measures anxiety states or traits. Walz et al. (2016) implemented the paradigm in an outdoor football field and found that, relative to healthy controls, individuals with agoraphobia and those with high anxiety sensitivity exhibited stronger thigmotaxis, fewer center entries, and longer latencies to the first center entry. These results are consistent with animal studies. However, Gromer et al. (2021) found inconsistent and weak correlations between thigmotaxis-related behaviors and multiple anxiety sensitivity scales (STAI, ASI-3, ACQ, MI), none of which remained significant after correction for multiple comparisons, although the majority of coefficients pointed in the direction of greater center avoidance with higher anxiety. Gromer et al. (2021) argued that this discrepancy may reflect the fact that, unlike animals directly facing predatory risk, humans perceive relatively little threat in open-field contexts, thereby limiting the paradigm’s sensitivity in general populations and restricting observable effects to the extremes of anxiety. Importantly, both studies focused only on behavioral–self-report associations, with no integration of physiological or neural data, indicating insufficient multimodal evidence at the construct level and limiting the paradigm’s translational validity.

At the applied functionality level, no pharmacological studies have yet been conducted, leaving predictive validity untested. In terms of discriminant validity, Walz et al. (2016) demonstrated that the human OFT can distinguish individuals with agoraphobia from healthy participants, as well as those with high versus low anxiety sensitivity. Behavioral indices such as wall-avoidance duration, number of center entries, and latency to first center entry showed significant differences, with medium-to-large effect sizes (Cohen’s d ≈ 0.7–1.1). However, these findings are based on small sample sizes and have not been extended to other clinical subtypes, leaving its diagnostic potential uncertain.

In summary, the human OFT, as a low-interference and ecologically valid measure of avoidance behavior, shows advantages in task design and in eliciting spontaneous behavioral responses. It also demonstrates preliminary discriminant potential for identifying high-anxiety-sensitivity individuals and patients with agoraphobia. However, its construct validity remains insufficiently established, with little supporting evidence from self-report, physiological, or neural measures, and its predictive validity has not been verified in pharmacological studies. Future research should systematically evaluate the OFT under conditions of heightened threat and across different anxiety subtypes, incorporating multimodal indicators to provide a more comprehensive validation of its utility in human anxiety research.

### 4.3. Validity of the Morris Water Maze

Human adaptations of the Morris Water Maze have primarily focused on spatial navigation and strategy use (Schoenfeld et al., 2017). At the experimental design level, the task is well standardized, procedurally straightforward, and demonstrates good feasibility. However, as a paradigm for assessing anxiety, its construct validity remains unclear. Kallai et al. (2005) and Kallai et al. (2007) reported that some individuals’ reliance on thigmotaxis during the initial phase of the task correlated with fear scores, but showed no stable association with state or trait anxiety. Moreover, these behavioral responses appeared to be more strongly influenced by cognitive ability, preventing the integration of behavioral, subjective, and physiological indices into a coherent construct of anxiety. To date, human MWM studies have not incorporated physiological or neural indices alongside behavioral and subjective data, and thus lack multimodal convergence at the construct level, which constrains their translational validity.

At the applied functionality level, no studies have yet employed pharmacological interventions or state manipulations to test the paradigm’s predictive validity, nor has its discriminant utility in clinical populations been systematically examined.

In summary, the MWM appears more suitable for evaluating spatial abilities than for directly measuring anxiety states or traits. Nevertheless, it may serve as a supplementary tool for examining the relationship between thigmotaxis and anxiety-related tendencies.

### 4.4. Validity of the Elevated Plus Maze

At the experimental design level, the human adaptation of the elevated plus maze demonstrates high operability and reproducibility. In terms of operability, the paradigm is highly standardized, requires equipment of moderate cost, and allows for the simultaneous collection of behavioral, autonomic, and endocrine measures, while the total testing time can be kept under ten minutes, facilitating its use in both healthy and clinical populations. For example, Biedermann et al. (2017) combined the paradigm with virtual reality to enhance ecological validity while ensuring participants’ physical safety. In terms of reproducibility, Biedermann et al. (2017) and Biedermann et al. (2024) examined the task across multiple virtual contexts (valley, seaside, desert, and gaming environments) and conducted a within-subject retest design. In the latter study, 44 healthy participants (19 females, 20 males) completed the task twice with a 28-day retest interval. Test–retest reliability was evaluated using intra-class correlations with a two-way mixed-effects model, consistency type (ICC (3,1)), appropriate for fixed experimental conditions. All ICC values for key behavioral measures exceeded the commonly accepted threshold for sufficient reliability: entries = 0.72 [95% CI = 0.46–0.85], latency = 0.71 [95% CI = 0.45–0.85], latency-end = 0.72 [95% CI = 0.46–0.85], time on open arms = 0.80 [95% CI = 0.62–0.89], total distance = 0.84 [95% CI = 0.69–0.92], and velocity = 0.79 [95% CI = 0.60–0.89].

These findings collectively demonstrate that the human EPM yields consistent behavioral outcomes across different visual contexts and retest sessions, confirming its within-subject reproducibility under standardized conditions. However, it should be noted that all existing reliability evidence has been obtained from healthy samples, and validation in clinical populations remains an important direction for future research.

At the construct measurement level, existing research provides preliminary evidence from physiological responses, behavioral performance, and subjective reports. First, participants exhibited accelerated heart rate, increased respiratory rate, and heightened skin conductance when approaching the open arm area, indicating significant autonomic nervous system activation in response to perceived threat. Second, at the endocrine level, salivary α-amylase rapidly increased after task completion, reflecting a swift sympathetic nervous system response. Cortisol levels peaked approximately 15 min later, indicating activation of the hypothalamic–pituitary–adrenal axis and continued processing and regulation of the threatening situation even after the task concluded. These physiological responses showed high consistency with behavioral indicators such as time spent in the open arms and number of entries, and exhibited a significant positive correlation with subjective anxiety ratings. This demonstrates that the paradigm can induce physiological states approximating real anxiety in non-threatening conditions and possesses certain anxiety measurement capabilities. However, no studies have directly measured the neural mechanisms underlying the behaviors induced by this paradigm, and neuroconceptual evidence remains to be supplemented.

At the applied functionality level, pharmacological studies have provided preliminary evidence for predictive validity: In Study 2 of Biedermann et al. (2017), a placebo-controlled, double-blind design was employed in which healthy participants received lorazepam (1 mg), yohimbine (20 mg), or placebo. The results showed a clear bidirectional pharmacological effect: lorazepam significantly increased time spent on open arms and reduced self-reported anxiety, whereas yohimbine decreased open-arm exploration and tended to elevate subjective anxiety. These findings demonstrate that the human mixed-reality EPM is pharmacologically sensitive to both anxiolytic and anxiogenic manipulation, consistent with results observed in animal models (Biedermann et al., 2017). Regarding discriminant validity, among healthy participants, those with higher self-reported anxiety explored the open arms less frequently and for shorter durations than those with lower anxiety, indicating that the paradigm can distinguish between different anxiety tendencies within non-clinical populations. However, it has not yet demonstrated diagnostic discrimination, and its applicability requires further validation in clinically diagnosed anxiety samples and subtypes.

In summary, the human EPM demonstrates strong operability and reproducibility. At the construct measurement level, initial support has been provided by behavioral, physiological, and subjective indices, though direct neural evidence is lacking. At the applied functionality level, the paradigm has been used in pharmacological studies and shows preliminary capacity to differentiate individuals with varying anxiety levels. However, its effectiveness in identifying clinical anxiety disorders or distinguishing among subtypes remains unclear. Future research should systematically examine its performance in clinical samples, integrating behavioral, subjective, physiological, and neural measures to clarify the validity and limitations of the paradigm in human anxiety research.

## 5. Future Directions

Human translational paradigms for anxiety have made initial progress in paradigm construction, empirical validation, and cross-species mapping. However, they remain in the early stages of exploration. Current limitations include insufficient systematicity in construct measurement, limited ecological validity of experimental contexts, and underdeveloped applications for clinical and research purposes (Table 2). To address these shortcomings, future research should advance along several key directions.

### 5.1. Strengthening Construct Validity of Anxiety Paradigms

To advance human anxiety paradigms from mere representational mimicry toward comprehensive mechanism construction and clinical translation, future research should systematically strengthen their performance in terms of construct validity, predictive validity, and discriminant validity.

First, regarding construct validity, most existing paradigms overemphasize behavioral indices while lacking integrated validation across subjective experience, physiological responses, and neural mechanisms. Future studies should collect multimodal data concurrently during tasks—including skin conductance, heart rate variability, self-report measures, and neuroimaging (fMRI, EEG)—to build “behavior–subjective–physiological/neural” pathway models, thereby enhancing the interpretive power of construct validity.

Second, in terms of predictive validity, only the human elevated plus maze has provided preliminary evidence of pharmacological sensitivity, while most other paradigms have not been examined through drug manipulations or state induction. Future research should employ interventions such as threat-of-shock paradigms (Gromer et al., 2021) or double-blind pharmacological trials to determine the predictive capacity of these tasks for anxiety. However, the implementation of pharmacological manipulations in human paradigms remains challenging due to ethical restrictions, methodological heterogeneity, and individual variability, resulting in limited pharmacological evidence and constraining the systematic evaluation of predictive validity (Cryan & Sweeney, 2011; Lonsdorf et al., 2017; Galderisi et al., 2024).

Finally, with respect to discriminant validity, most current studies rely on healthy participants or healthy participants stratified by self-reported anxiety levels. To date, only the open-field test has been applied to clinical populations, showing that agoraphobic patients exhibited pronounced avoidance behavior (Walz et al., 2016). However, no paradigm has yet been systematically compared across distinct anxiety disorder subtypes, such as generalized anxiety disorder or social anxiety disorder. Future research should incorporate clinical samples, employ standardized diagnostic tools (e.g., DSM-based assessments), and integrate multimodal indices to clarify the appropriateness of each paradigm for subtype identification and individualized prediction.

In addition, recent advances in computational modeling enable researchers to investigate the mechanisms underlying observable behaviors. For instance, Piray and Daw (2021) demonstrated that anxious individuals tend to overestimate environmental volatility while underestimating randomness. In the future, integrating such modeling approaches with behavioral paradigms could further elucidate the mechanisms that drive anxiety-related behaviors (Piray & Daw, 2021).

### 5.2. Integrating Virtual Reality for Complex Anxiety Scenarios

Most current human translational paradigms for anxiety are adapted from animal models, primarily simulating avoidance responses to spatial threats, uncertainty, or heights. However, human experiences of anxiety are more frequently driven by complex social interactions and higher-order cognitive processes. As a result, mechanisms commonly used to induce anxiety in animals may elicit only mild responses in humans, often leading to weak associations between behavioral performance and subjective reports, thereby undermining the construction of construct validity.

To enhance both construct activation and ecological validity, future research should develop experimental scenarios that integrate social and cognitive complexity, explicitly incorporating uniquely human features of emotional processing into paradigm design. A recently developed tool, the VRThreat Toolkit (Brookes et al., 2024), provides important technological support for this endeavor. The toolkit integrates a range of natural and social threat scenarios (e.g., predatory animals, environmental disasters, human aggressors) along with dynamic interaction mechanisms (e.g., path selection, behavioral feedback, motion capture). It enables the elicitation of realistic defensive responses in a controlled and ethically safe environment, while simultaneously recording behavioral, physiological, and subjective data. Furthermore, it can be synchronized with EEG and wearable devices, offering a feasible pathway toward the development of ecologically valid, high-resolution paradigms for measuring anxiety. Beyond spatial and physical threats, these VR-based methods could further be extended to social contexts, as virtual reality exposure therapy has shown strong and clinically comparable efficacy to traditional exposure treatments for social anxiety (Powers & Emmelkamp, 2008; Horigome et al., 2020), thereby enhancing both ecological validity and clinical relevance.

### 5.3. Leveraging Wearable Devices for Real-Time Monitoring

Traditional anxiety measurement methods such as questionnaires and interviews are predominantly static and retrospective, making it difficult to capture the dynamic fluctuations and immediate physiological changes associated with anxiety states. With the widespread adoption of smart wearable devices and behavioral sensing technologies, anxiety states may now be converted into data patterns that can be tracked and quantified in real time.

Currently, mainstream smart wearables on the market can estimate an individual’s stress levels by measuring physiological signals such as heart rate variability, skin conductance, skin temperature, and respiratory rate. These devices assist wearers in daily emotional management and enable real-time self-intervention. Future research could collect behavioral data reflecting anxiety—such as gait changes, activity range, spatial trajectories, and interaction patterns—alongside synchronized physiological data. Coupled with machine learning models, these multimodal data could support the development of individualized anxiety-recognition systems.

Once such data are structured, quantified, and accumulated over time, they could enable real-time detection, trend forecasting, and behavioral alerts for anxiety. When anxiety is no longer conceptualized solely as a subjective experience but as a dynamically computable state variable, it will be possible to shift from language-based descriptions to data-driven assessments, and from discrete measurements to continuous process monitoring. This transformation holds promise for advancing early screening, personalized interventions, and long-term outcome tracking.

## 6. Conclusions

This review has systematically examined the translation of animal anxiety paradigms into human experimental tasks, outlining the theoretical logic, design features, measurement indices, and application potential of representative paradigms. It proposes a “three-level, five-dimension” validity framework to evaluate the usability and limitations of human translational tasks. Although preliminary progress has been made, the field remains in an exploratory stage, facing challenges such as limited ecological scope, insufficient construct validity, and unclear predictive power. Future research should aim to enhance ecological realism, integrate multimodal indicators, and include clinical samples, thereby laying the groundwork for more sensitive, valid, and clinically applicable paradigms for the assessment of anxiety.

## Figures and Tables

**Table 1:** Animal anxiety models and their human translational tasks.

Model Type	Animal Representative Paradigms	Conflict Type	Key Behavioral Indices	Human Translational Paradigms
Conditioned conflict paradigms	Vogel test, Geller–Seifter test	Conflict between reward-seeking and punishment avoidance	Frequency of punished drinking/feeding responses	Approach–Avoidance Conflict task
Unconditioned conflict paradigms	Open field test	Conflict between exploration and avoidance of open/novel spaces	Time spent in center; exploratory approach behaviors	Virtual or real open-field task
	Morris water maze	Conflict between exploration and avoidance of open/novel water environments	Navigation strategies; latency/path to hidden platform	Virtual or real water-maze task
	Elevated plus maze	Conflict between exploration and avoidance of elevated open spaces	Open-arm entries; time spent in open arms	Virtual elevated maze task

**Table 2:** Evaluation of human translational paradigms of anxiety under the “three-level, five-dimension” validity framework.

Model Type	Experimental Design		Construct Measurement	Applied Functionality	
	Operability	Repeatability		Predictive Validity	Discriminant Validity
Approach–Avoidance Conflict Paradigm	High	Behaviorally replicated; quantitative reliability evidence lacking	Evidence from behavioral and neural measures; lacking evidence from self-report and physiological indices	Not examined	Able to distinguish only sex differences
The Open Field	High	Behaviorally replicated; quantitative reliability evidence lacking	Evidence limited to behavior only	Not examined	Can differentiate patients with agoraphobia and health individuals with high vs. low anxiety sensitivity
Morris Water Maze	High	Behaviorally replicated; quantitative reliability evidence lacking	Evidence limited to behavior only	Not examined	Not examined
Elevated Plus Maze	High	High (ICC > 0.7)	Evidence from behavioral, physiological, and self-report measures; lacking neural evidence	Tested the effects of the anxiolytic lorazepam and the anxiogenic yohimbine	Can differentiate health individuals with different anxiety levels

## Data Availability

Data sharing is not applicable. No new data were created or analyzed in this study.
